# Alveolar rhabdomyosarcoma with massive bone marrow infiltration and disseminated intravascular coagulation mimicking acute leukemia

**DOI:** 10.1002/jha2.447

**Published:** 2022-05-18

**Authors:** Ayano Sugihara, Hiroo Katsuya, Hiroshi Ureshino, Shinichi Kido, Shinya Kimura

**Affiliations:** ^1^ Division of Hematology Respiratory Medicine and Oncology Department of Internal Medicine Faculty of Medicine Saga University Saga Japan; ^2^ Division of Pathology and microbiology Faculty of Medicine Saga University Saga Japan

1

A 27‐year‐old man was admitted with a 2‐month history of general fatigue, appetite loss, and back pain, leading to a worsening of his general condition. On admission, laboratory tests showed an increased white blood cell count (15.9 × 10^9^/L), with leucoerythroblastosis (myeloblasts, 1.0%; promyelocytes, 0%; myelocytes, 2.5%; metamyelocytes, 1.5%; band neutrophils, 3.5%; segmented neutrophils, 67.0%; lymphocytes, 18%; erythroblasts, 48%), severe anemia (hemoglobin, 45.0 g/L), and thrombocytopenia (platelet count, 21.0 × 10^9^/L). Biochemical tests revealed elevated lactate dehydrogenase (2582 U/L) level, and coagulation tests showed a prolonged prothrombin time (20.3 s; reference interval: 10.0–13.0), normal fibrinogen (2.24 g/L), elevated fibrinogen‐fibrin degradation product (331.9 mg/L), plasmin inhibitor‐plasmin complex (9.05 mg/L), thrombin‐antithrombin complex (41.2 μg/L), and decreased antithrombin (75.3%) levels. A computed tomography scan revealed systemic lymphadenopathy, splenomegaly, bilateral pleural effusions, and massive ascites. A bone marrow aspirate revealed massive infiltration by large abnormal cells with a basophilic cytoplasm and cytoplasmic vacuoles (Figure 1A). Flow cytometry analysis showed that the abnormal cells expressed only CD56, but no myeloid or lymphoid cell markers. Because he suffered severe disseminated intravascular coagulation (DIC) and worsening hypoxemia due to increased pleural effusions, initial treatment for acute leukemia comprised prednisolone and cytarabine. On Day 2 postadmission, biopsy of the right axillary lymph node was performed. Immunohistological findings from a bone marrow clot and biopsy tissue from the right axillary lymph node revealed diffuse proliferation of abnormal round cells, which expressed desmin (Figure 1B), myogenin, vimentin, and CD56 (Figure 1C), but not cytokeratin and leukocyte antigen. Therefore, a diagnosis of alveolar rhabdomyosarcoma was made. Subsequently, treatment with vincristine, doxorubicin, and cyclophosphamide was initiated, which ameliorated the lymphadenopathy, ascites, and DIC.

**FIGURE 1:**
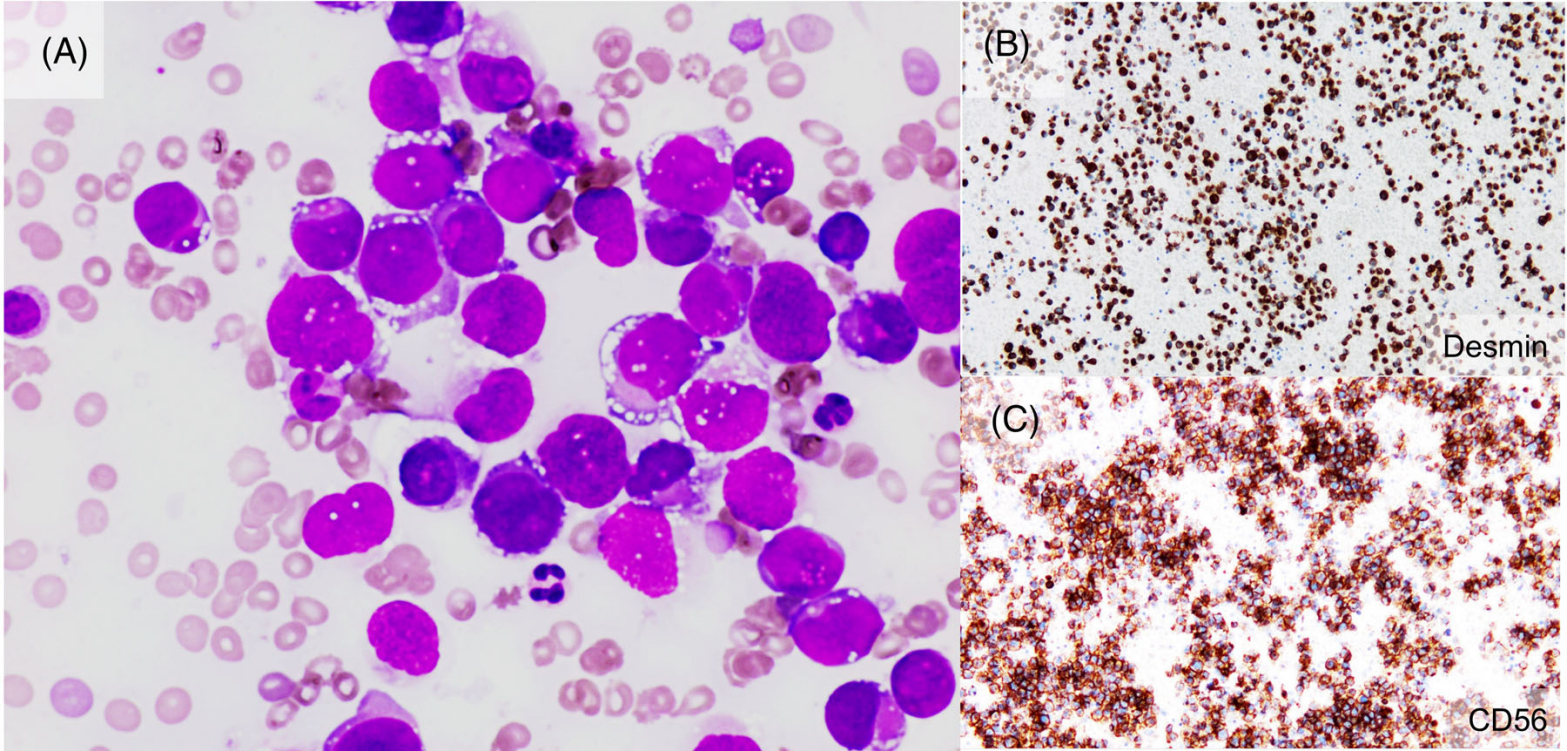
(A) Bone marrow aspirate (original magnification, ×400; May–Giemsa staining) shows large abnormal cells with basophilic cytoplasm and cytoplasmic vacuoles. Immunohistological analysis revealed that the bone marrow clot was positive for desmin (B) and CD56 (C) (original magnification, ×60; (B) and (C))

Although a leukemic phase or bone marrow infiltration is an uncommon manifestation of alveolar rhabdomyosarcoma, flow cytometry and immunohistological analyses of bone marrow specimens can facilitate correct diagnosis.

## CONFLICT OF INTEREST

The authors declare that there is no conflict of interest that could be perceived as prejudicing the impartiality of the research reported.

## FUNDING

The authors received no specific funding for this work.

## AUTHOR CONTRIBUTIONS

AS, HK, HU, and SK contributed to patient's care. SK performed pathological diagnosis. All authors wrote and reviewed the manuscript. Written informed consent for publication was obtained from the patient.

## ETHICS STATEMENT

All procedures performed in studies involving human participants were in accordance with the ethical standards of the institutional and/or national research committee and with the 1964 Helsinki declaration and its later amendments or comparable ethical standards. Written informed consent for publication was obtained from the patient.

## Data Availability

All data can be assessed by contacting the corresponding author.

